# Deception Affects Interbrain Electroencephalographic and Autonomic Synchronization Within a Dyad: A Hyperscanning Study

**DOI:** 10.1111/nyas.70217

**Published:** 2026-02-11

**Authors:** Giorgio Veneziani, Federica Luciani, Emanuele Giraldi, Virginia Campedelli, Carlo Lai

**Affiliations:** ^1^ Department of Dynamic and Clinical Psychology, and Health Studies, Faculty of Medicine and Psychology Sapienza University of Rome Rome Italy

**Keywords:** deception, EEG, heartbeats, hyperscanning, interbrain synchronization

## Abstract

Research on deception has focused on the neurophysiological assessment of the deceiver, showing activation of specific brain areas and increased autonomic activity. However, deception is an interpersonal process where both the deceiver and the deceived interact in a constant process of evaluation that requires demanding cognitive resources. The present study aimed to investigate interbrain synchronization (IBS) and heart rate synchrony between an interviewer intent on detecting deception and an interviewee during a deception (deception group, or DG) or truth‐telling (non‐deception group, or NDG) task using an ecological mock crime experiment. The results showed that DG exhibited higher IBS before the interview in the theta band and during the interview in the alpha band, while displaying decreased heart rate synchrony in the high frequency band compared to NDG. The greater IBS in DG involved, particularly, the left temporal area of the interviewee. These findings highlight the relevance of studying deception according to a two‐person neuroscience perspective, suggesting that while neural processes are synchronized before and during a deceptive interaction, autonomic processes follow different activation patterns. Integrating the hyperscanning techniques with existing lie‐detection methods could enhance the identification of neurophysiological markers of deception.

## Graphical abstract

The deception group (DG) exhibited higher inter‐brain synchronization (IBS) before the interview in a theta band cluster and during the interview in an alpha band cluster, while displaying decreased heart rate synchrony (HRS) in the high frequency band compared to the non‐deception group (NDG). The findings highlight the importance of a two‐person neuroscience perspective in identifying neurophysiological markers of deception.

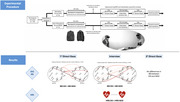

## 1 Introduction

Deception is a process in which a deceiver convinces others to accept a belief or interpretation, conveying false information [1−4]. The act of deceiving involves both verbal and nonverbal efforts, where deceivers tend to experience an increase in general arousal, emotion, and cognitive load, and a tendency to manage their image to maintain credibility [1, 5−8]. The higher cognitive demands associated with deception, compared to telling the truth, have been highlighted by previous research, showing that deception can lead to experiencing negative affect, probably due to violating social norms [1, 6, 8]. Considering its significant role in criminology and forensic psychology, deception has garnered considerable attention in recent years from the literature [4, 9−12].

As a complex social interaction, deception involves several brain areas related to executive functions [13, 14]. Previous studies showed increased activity in brain areas involved in high cognitive processes, such as mentalizing, behavioral inhibition, and decision‐making, during deception tasks [4, 15, 16]. In particular, the involvement of the prefrontal cortex and temporal lobe was suggested in the intentional falsification processes and deception [11, 17−21]. Several studies using event‐related potential advised the efficiency of electroencephalogram (EEG) in identifying detection [2, 22, 23]. However, the brain connectivity patterns and mechanisms underlying deception were less investigated [24−27], despite phase oscillatory activity appearing to be an efficacious neural correlate of many high cognitive processes involved in deception [25, 28]. Moreover, autonomic system activations, particularly at the cardiovascular level, were found to be effective physiological signals for recognizing deceptive situations [29−31]. In this regard, deceptive behavior was shown to be associated with increased heart rate, which could reflect a higher physiological arousal and cognitive load in generating deceptive information [29]. Furthermore, previous studies found that increased cardiovascular activity may indicate the level of stress in immoral situations [32, 33]. Accordingly, deception seems to involve both cognitive and emotion‐related response patterns, making the combination of neural and autonomic measurements a useful and effective approach to improve the accuracy of detecting deception [29, 34].

In addition, it is important to consider that deception is an interpersonal process in which both deception and its detection are associated with individuals’ arousal, negative affects, cognitive tension, and attempts to control [1, 35]. In this regard, despite numerous individual‐focused brain imaging studies being conducted, it could be useful to examine deception in the context of the dynamic interaction between individuals face‐to‐face [4, 36]. Indeed, several nonverbal cues, such as tone of voice and eye contact, play a pivotal role in deciphering others’ intentions and mental states [37, 38], whereby it was shown to be associated with activations of brain areas related to social cognition [39]. In the presence of another person, deceivers have to produce an appropriate deceptive message, controlling their behavior, avoiding cues of deception (such as changes in tone of voice and speech rhythm), and appearing natural according to the other person's reactions [8, 36]. At the same time, the individual trying to recognize deception can identify relevant nonverbal cues in the behavior and interpret them to determine whether the other person is deceiving or telling the truth [36].

Studies using the hyperscanning technique appear to offer interesting new insights into understanding the neural correlates involved in social interactions [40−44]. This technique involves simultaneously measuring the neural activities of multiple individuals during interactive tasks [45]. Research within this theoretical framework considers the two brains as a single system, evaluating the associations between regions of two or more brains [46] through interbrain synchronization (IBS) [47]. Several studies showed that specific interpersonal processes were associated with IBS at different frequencies [48−50]. In particular, coordinated behaviors were found to be associated with higher IBS in the theta band (4−7 Hz) [48, 51], suggesting its potential role as a marker of social cognition [52]. Theta and alpha (8−12 Hz) IBS seem particularly sensitive to human contact, with greater theta and alpha IBS observed in human−human interactions compared to human−computer interactions [53]. Moreover, some studies found an association between affective attentional mechanisms with the alpha rhythm [54], suggesting its role in maintaining attention to environmental, emotionally salient stimuli [55]. The hyperscanning technique has been extended to the study of complex interpersonal phenomena, including deception, a process that inherently involves a mutual interpretation of intentions and management of beliefs [4, 10, 36]. In this regard, using functional near‐infrared spectroscopy (fNIRS), it was shown that deception was associated with increased IBS in the left posterior superior temporal sulcus, suggesting how this brain region may play a pivotal role in deceptive acts due to mentalization requirements related to modulation of others’ thoughts [4]. In addition, a further study examined spontaneous face‐to‐face deception using an fNIRS hyperscanning paradigm, showing increased IBS during deception tasks in the prefrontal cortex in female dyads and in the temporal parietal junction in male dyads [10]. Taken together, these results highlight that deception would appear to be associated with synchronization in frontal, temporal, and parietal areas, suggesting that deceptive processes might be supported by interactive neural dynamics, a probable correlate of a bidirectional communicative process between the deceiver and the deceived.

Interestingly, socioemotional interactions between two individuals were also associated with the synchrony of their heart rates [56−58], suggesting that synchronization can occur at both the neural and autonomic levels during these interactions. In particular, it was found that heart rate synchrony increased during cooperative tasks [56, 59] and during interactions aimed at eliciting a shared emotional arousal in romantic partners [57], while decreasing in competitive contexts [60]. Moreover, previous studies showed that heart rate synchrony varied according to emotional closeness, finding increased levels of synchrony associated with the degree of emotional bonding between pairs of participants and in those who had a close working relationship or lived together in a bonding relationship [61−63].

The only two studies conducted in the literature that used the hyperscanning technique to investigate spontaneous deception employed a card‐gambling paradigm [4] and a sender‐receiver paradigm based on a game‐theoretic modeling task [10] in which both experimental conditions left it up to the participants to decide when to deceive. Although these studies used ecological and naturalistic experimental paradigms where deception occurs, they represent game‐based laboratory interactions that differ substantially from real‐world forensic contexts in which individuals must actively manage reactions, conceal intentions, and answer questions under social pressure to successfully deceive another person. Accordingly, the present study adopts a mock crime paradigm with a structured interview setting by manipulating the deception condition that reflects more closely the forensic dynamics of an interrogation, in which interviewees must strategically manage verbal and nonverbal behavior to appear trustworthy. In addition, to further delve into the neurophysiological mechanisms underlying deceptive behavior, the study investigates both neural and autonomic synchronization during deception. Highlighting this interpersonal dynamic could inform the development of more refined models of deception detection in forensic psychology, moving beyond approaches that focus solely on isolated behavioral cues or the activity of a single individual.

Therefore, the present study aimed to investigate EEG IBS and heart rate synchronization between an interviewer intent on detecting deception and an interviewee during a deception or truth‐telling task using an ecological mock crime experiment. In particular, differences in neural and heart rate synchronization (interviewer−interviewee) were evaluated between a group in which the interviewee deceived (deception group, “DG”) and a group in which the interviewee did not deceive (non‐deception group, “NDG”). The hypothesis was that DG would show increased neural synchronization compared to NDG. Regarding autonomic synchronization, no specific hypotheses were formulated, in accordance with an exploratory aim.

## 2 Materials and Methods

### 2.1 Participants

Thirty healthy right‐handed individuals with normal or corrected‐to‐normal vision (16 females; *M_age_* = 25.2 years, *SD_age_* = 3.7 years; range = 20–34 years) were included in the present study. The sample was determined on the basis of previous EEG hyperscanning studies, such as Balconi and Vanutelli (*N* = 30) [52] (*N* = 8) [64], Toppi et al. (*N* = 12) [65], Veneziani et al. (*N* = 18) [66], Moreau et al. (*N* = 20) [67], and Daffinà et al. (*N* = 30) [68]. All participants self‐reported that they did not take medication and did not have past or present neuropsychiatric, neurological disorders, and health problems (such as head injuries). All participants were informed about the purpose and procedure of the experiment and gave their written informed consent prior to participation. The present study was conducted in accordance with the Declaration of Helsinki (1964) and was reviewed and approved by the Ethics Committee of the Department of Dynamic and Clinical Psychology, and Health Studies, Sapienza University (protocol number: 0000589).

### 2.2 Group Assignment

Participants were randomly assigned to the deception group (DG; *n* = 15; 7 females; *M_age_* = 26.2 years, *SD_age_* = 3.9 years; range = 22–34 years) or the non‐deception group (NDG; *n* = 15; 9 females; *M_age_* = 24.1 years, *SD_age_* = 3.1 years; range = 20–32 years). In the DG, participants were instructed to lie about the contents of a backpack (as described in the following paragraph), while in the NDG, participants were instructed to tell the truth about its contents during an interview they would undergo with an interviewer.

### 2.3 Backpack

An experimenter provided each participant with an empty backpack to pack specific items. Participants in both groups (DG and NDG) filled their backpacks with items habitually possessed by visitors to the Department (“nonrestricted”) (personal computer, sunglasses, t‐shirt, box of painkillers, and USB flash drive) and items of uncommon use and considered dangerous (“restricted”) (bottle of alcohol, hammer, knife, lighter fluid, and legal cannabis).

### 2.4 Interview

The interview was designed to obtain verbal responses regarding the content of the participants’ backpacks. In particular, a structured interview (Table S1), adapted from Mapala and colleagues [69], consisting of 30 yes/no questions in random order written on paper, was used. Ten questions evaluated the presence of nonrestricted items (e.g., sunglasses, notebook, etc.), 10 assessed the presence of restricted items (e.g., alcohol, hammer, etc.), and 10 were unexpected questions about general information (e.g., “Do you think there is traffic today?”; “Is today your birthday?”).

The interview was conducted by a recruited interviewer (male, 26 years old, PhD student) who signed the informed consent and was instructed to identify whether the interviewee lied or did not lie about the backpack's content. The interviewer was not affiliated with the research group and was not familiar with the research hypotheses and specific aims of the experiment. The interviewer's role was limited to administering the structured interview to the interviewees and maintaining a neutral and consistent communication style across the different experimental sessions. The interview room was set up with two chairs placed 2 m apart, with a table in the middle, on which the structured interview was placed, facing the interviewer. The study recruited only one interviewer to control for the potential influence that different interviewers could exert on the IBS, considering the high intersubjective variability to which EEG data are subject [70].

### 2.5 Procedure

**FIGURE 1:**
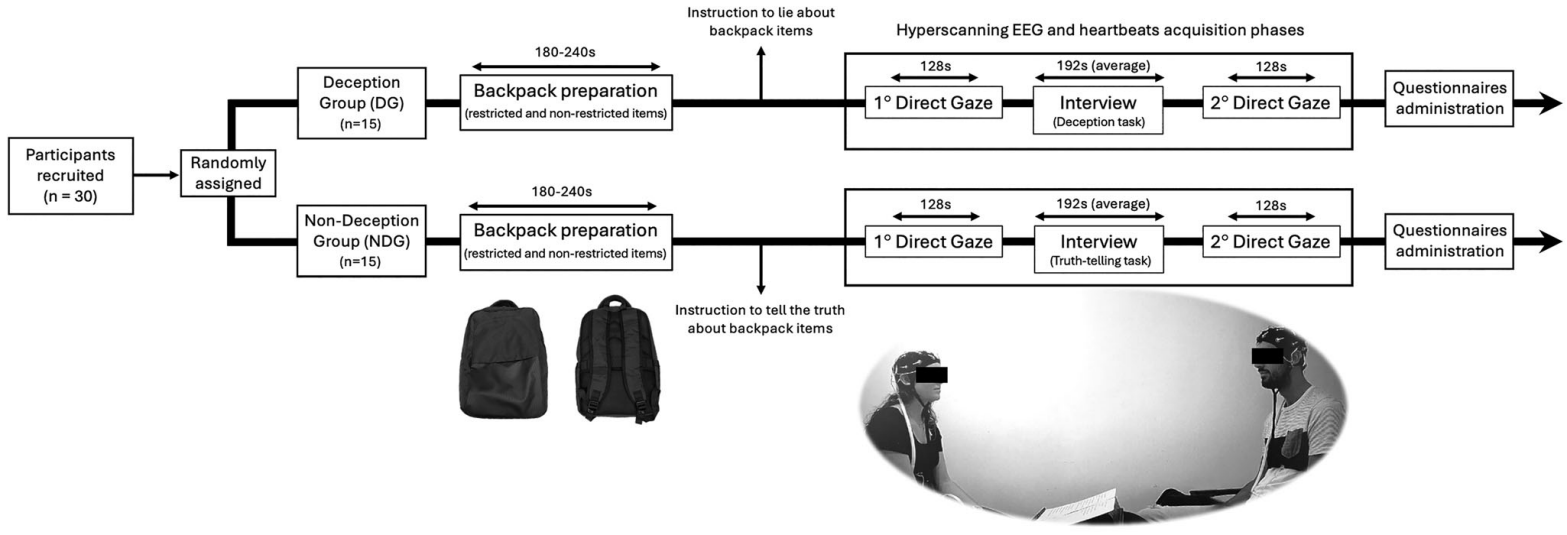
Schematic description of the experimental procedure. The first phase of the procedure consisted of randomly assigning participants to either the deception group (DG) or the non‐deception group (NDG). Subsequently, participants filled the backpack with the items provided and were instructed to lie (DG) or tell the truth (NDG) about its contents. Afterward, the hyperscanning EEG and heartbeats acquisition phases began between the interviewer and the participant. Initially, each dyad was asked to maintain eye contact for 128 s (First Direct Gaze) before the interview started. Then, the interviewer administered the structured interview to the participant (Interview; mean duration 192 s). Finally, after the interview, each dyad was asked to make eye contact for 128 s (Second Direct Gaze). Participants were administered demographic and psychological questionnaires at the end of the experimental procedure.

The research was conducted at the Department of Dynamic and Clinical Psychology and Health Studies at Sapienza University of Rome. The interviewer arrived about 5 min before the participant and was accompanied by an experimenter to the interview room. The participant, upon arrival, signed the informed consent and was randomly assigned to either DG or NDG (Figure 1). Subsequently, the participant filled the backpack with the provided items and was instructed to lie (DG) or tell the truth (NDG) about its contents during the interview that would follow. Afterward, the participant was accompanied to the interview room, where they joined the interviewer and where both were fitted with the EEG and heartbeats acquisition system. To maintain the unfamiliarity between the interviewer and the participant, they were seated beside each other with a dividing panel and were guided not to speak [71]. Once the sensors were set up, the experimenter removed the dividing panel and, before leaving the room, asked the dyad to maintain eye contact for a short period of time, limiting body movements as much as possible. The hyperscanning EEG and heartbeats acquisition phases between the interviewer and the participant then started. After 128 s of direct gaze (First Direct Gaze), the experimenter re‐entered the room and instructed the interview phase (Interview), which involved administering the structured interview, asking the interviewer and the participant to speak slowly and maintain eye contact with each other. During this phase, the interviewer only moved his eyes to read each question and then repeated it while staring at the participant. At the end of the interview, after about 3 min, the experimenter re‐entered the room, giving instructions for the last phase, in which the interviewer and the participant were asked to maintain eye contact (Second Direct Gaze). At the end of this phase, which lasted 128 s, the experimenter re‐entered the room and asked the participant to leave. The participant was then taken to another room to complete the demographic and psychological questionnaires. Meanwhile, the experimenter asked the interviewer to provide a judgment on whether the participant had lied regarding the items in the backpack.

Encephalan‐EEGR software (Medikom MTD, Russia) was used to control the timing and to video record all the experimental phases.

### 2.6 Questionnaires

Information was collected regarding gender, age, civil and professional status, education level, and motivation to participate in the study. In addition, the Personality Inventory for DSM‐5‐Brief Form (PID‐5‐BF) [72] was used in the present study to assess personality dimensions as characterized by DSM‐5. Indeed, considering that previous research has shown how different personality traits modulate neural activities [73], the present study used the questionnaire to investigate differences between the DG and NDG in maladaptive personality traits. The PID‐5‐BF was developed by extracting 25 items from the original PID‐5, representing 21 of the 25 trait facets. Items are rated on a 0–3 Likert‐type scale, with higher scores representing more significant dysfunction. Each of the five higher‐order domains is represented by five items (Negative Affect: items 8, 9, 10, 11, and 15; Detachment: items 4, 13, 14, 16, and 18; Antagonism: items 17, 19, 20, 22, and 25; Disinhibition: items 1, 2, 3, 5, and 6; and Psychoticism: items 7, 12, 21, 23, and 24).

### 2.7 EEG and Heartbeats Acquisition and Preprocessing

The Encephalan Main Syncro EEG system comprises two units, featuring synchronous acquisition and video recording of the participants’ neural activities (Medikom MTD). The system comprehended two caps with 19 electrodes arranged according to the international 10/20 system (Fp1, Fp2, F7, F3, Fz, F4, F8, T3, C3, Cz, C4, T4, T5, P3, Pz, P4, T6, O1, O2), the neutral (N), and the two references electrodes (A1 and A2). The caps were aligned to nasion, inion, and left and right preauricular points. Two electrooculograms (EOGs) and one electromyogram (EMG) were used to record the ocular and muscular artifacts. In addition, one electrocardiogram (ECG) was used to acquire the cardiac artifacts and the number of heartbeats. The impedances were maintained below 5 kΩ. The signals were online filtered between 0.5 and 70 Hz, and the sampling frequency was 250 Hz.

An automatic algorithm implemented in Encephalan‐EEGR software was used to suppress blinking, eye motions, facial muscle activity, and cardiac activity [66, 74]. The detection and removal of EOG artifacts can be divided into three main steps: threshold setting, EOG detection, and EOG removal. The “threshold setting” algorithm is based on the standard deviation of the signal. Specifically, (1) the standard deviation (SD) of the signal is computed; (2) the difference between each value (v) and the mean value ($\bar{v})$ is computed; (3) if $|v-\bar{v}|>1.5*SD$, v is marked as an outlier and excluded from the subsequent calculations; (4) the standard deviation of the remaining values following the exclusion of outliers ($SD_{no\_outlier}$) is calculated; and (5) the threshold Th is computed as defined in Equation (1):

(1) $$Th=k*S\;D_{no\_outlier}=k*\sqrt{\frac{1}{N-1}\sum_{v\varepsilon L}{\left(v-\bar{v}\right)}^{2},}$$

where k is a settable parameter to make the algorithm more conservative or more tolerant, *v* is the value considered, *v̅* is the mean value of the time portion, *L* is the subset of values of the time portion following the exclusion of outliers, and *N* is the cardinality of *L*. Once the threshold Th is calculated, the “EOG detection” algorithm starts, which checks two conditions to identify where an artifact is present: (1) the value v must be greater than 10μV, and (2) $|v-\bar{v}|$ must be greater than the previously computed threshold Th. If both conditions are met, the EOG artifact is detected. To make the artifact detection algorithm more conservative, the coefficient *k* in Equation (1) was set to 0.8, allowing the second condition to be met more frequently.

Once the artifacts are detected, the software removes the EOG artifact (*X*) from the individual EEG channels (*Y*), according to the linear regression algorithm shown in Equation (2):

(2) $$Y^{\prime }=Y-mX,$$

where *Y′* is the EEG signal cleaned of the artifact, *Y* is the raw EEG signal affected by the artifact, *m* is the similarity coefficient between EOG and EEG, and *X* is the EOG signal acquired from the EOG electrode.

The EMG detection algorithm computes the difference between each value and the previous one. If the absolute value of the difference is greater than 5μV, the EMG algorithm identifies an EMG artifact and applies a linear regression such as the one in Equation (2).

Regarding cardiac artifacts on EEG signals, a dedicated electrode was used during the acquisition to record the ECG trace. The ECG artifacts detection and removal can be divided into five main steps: artifact identification on the ECG trace, epoching, phase shift computation, coefficient computation, and ECG removal. (1) The first step is a peak detection algorithm to identify the exact sample where the R‐peak of each heartbeat is $\left(R_{peakECG}\right)$. (2) The epoching procedure segments the signals into epochs, as illustrated in Equation (3):

(3) $$Epoc\;h_{i}=\left[R_{peakECG_{i}}-20;R_{peakECG_{i+1}}-20\right].$$

Since the sampling frequency is 250 Hz, each epoch goes from 80 ms before an R‐peak to 80 ms before the following R‐peak. (3) On each epoch, the phase‐shift is calculated to account for the temporal delay between the R‐peak detection of the ECG electrode and the corresponding ECG artifact on the EEG trace. To do so, the algorithm analyzes a temporal window around $R_{peakECG}$ and looks for the sample whose amplitude is maximum in the EEG trace $\left(R_{peakEEG}\right)$. (4) The coefficient K is calculated on each epoch by computing the ratio between the amplitude of $R_{peakEEG}$ and the amplitude of $R_{peakECG}$, as follows:

(4) $$K=\frac{v_{R_{peakEEG}}}{v_{R_{peakECG}}}.$$

(5) To correct the ECG artifact on the EEG trace, the two traces are aligned by compensating for the time shift, and the cleaned EEG is computed as follows:

(5) $$y_{EEG}^{\prime }=y_{EEG}-K*y_{ECG},$$

where $y_{EEG}^{\prime }$ is the cleaned EEG, $y_{EEG}$ is the EEG trace with ECG artifacts, K is the correction coefficient, and $y_{ECG}$ is the ECG trace.

Subsequently, the EEG data for each experimental phase (First Direct Gaze, Interview, and Second Direct Gaze) related to a dyad were exported to Python for further preprocessing using the open‐source library Hyperscanning Python Pipeline (HyPyP) [75]. Specifically, 1‐s epochs [76, 77] were created and further cleaned using a HyPyP function adapted from Autoreject [75, 78]. The function uses an algorithm with Bayesian optimization as the threshold method, interpolating data from bad sensors per participant. It rejects epochs containing transient spikes in isolated EEG electrodes and artifacts affecting multiple channels. The autoreject function employs the “union” strategy, which retains only electrodes and epochs deemed “good” for both participants, while immediately rejecting those considered “bad” for either subject. The rejected epochs for each phase are shown in Table S2.

### 2.8 EEG Hyperscanning Analyses

HyPyP was used to analyze interbrain activities [75]. Coherently with a previous hyperscanning study, the analytic signals were computed by applying infinite impulse response filtering and the Hilbert transform [79]. To evaluate the interbrain synchrony, the following frequency bands were considered: Theta (4−7 Hz) and Alpha (8−12 Hz). The phase locking value (PLV) for each frequency was calculated and averaged across the epochs of a specific experimental phase. The PLV measures interbrain synchrony by detecting the rhythmicity between the recorded EEG signals of two brains. This is a standard technique for analyzing the instantaneous phase of two signals in EEG hyperscanning studies and measuring the intratrial consistency of the phase difference between electrodes [80]. Specifically, the PLV measured in the present study is calculated by:

(6) $$\mathrm{PL}V_{t}=\frac{1}{T}\;\left|\sum_{n=1}^{T}e^{i(\phi_{\left(t,n\right)}\;-\;\psi_{\left(t,n\right)}}\right|,$$

where *T* is the number of time samples within the considered window, *e* is Euler's number, *i* is the complex operator, *ϕ* (*t*, *n*) corresponds to the phase on observation *n* at time *t* in channel *ϕ*, and *ψ* (*t*, *n*) corresponds to the phase on observation *n* at time *t* in channel *ψ*. The PLV*_t_* could vary between 0 (no phase locking over time) and 1 (perfect phase locking over time).

Lastly, shuffled pair analyses were conducted. Following procedures used in previous hyperscanning studies [48, 81−83], shuffled datasets were generated by randomly pairing interviewers and interviewees who did not perform the experimental procedure together, preserving group assignment (DG vs. NDG). Each shuffled dyad contained EEG signals recorded in the same experimental phase but from a different interviewer−interviewee dyad. Then, PLV was calculated for each shuffled dyad, following the same procedure used for the real dyads.

### 2.9 Heart Rate Coherence Analyses

ECG traces were exported at 250 Hz and analyzed in MATLAB (version R2022b) [84]. Initially, ECG traces were filtered with bandpass filtering (0.5−40 Hz) and QRS enhancement (5−15 Hz). R peaks were detected using an energy envelope approach, based on the squared first derivative followed by a 150 ms moving average, and a minimum time constraint of 0.3 s between consecutive peaks [85−87]. Subsequently, the interbeat interval series (RR, in seconds) was derived from consecutive R peaks, and intervals beyond 0.3−2.0 s were excluded [88, 89]. The instantaneous heart rate (HR) (in bpm) was then calculated as 60/RR and resampled to 4 Hz using piecewise cubic Hermite polynomials that preserves the signal's local shape [90, 91]. The HR series were then smoothed using the Savitzky−Golay filter (polynomial order 3 and frame length 61) [92, 93]. HR coherence was evaluated as the squared spectral coherence between HR series of dyads, using Welch's method (Hamming windows of 256 samples, 50% overlap, 512‐point fast Fourier transform) [94]. Coherence indices range between 0 and 1 values, where higher values indicate greater coherence of dyad HR signals. Specifically, average HR coherence was calculated in the low‐frequency (LF; 0.04−0.15 Hz) and high‐frequency (HF; 0.15−0.40 Hz) bands [92], yielding an LF and HF coherence index for each dyad and experimental phase.

### 2.10 Statistical Analysis

After the descriptive analyses, independent *t*‐tests were performed to evaluate the differences in age, education, and maladaptive personality traits between the DG and NDG using JASP software (v. 0.18.3) [95]. HyPyP was used to perform cluster‐level statistics provided by repeated‐measures analyses of variance (ANOVAs) and independent *t*‐tests [75]. Specifically, 3 × 2 repeated‐measures ANOVAs were conducted with the experimental phase (First Direct Gaze vs. Interview vs. Second Direct Gaze) as within‐subjects factor and group (DG vs. NDG) as between‐subjects factor for both Theta and Alpha bands. The same 3 × 2 repeated‐measures ANOVAs described for real dyads were then conducted on shuffled datasets. Subsequently, independent *t*‐tests were used to compare PLV values along the scalp between DG and NDG for each experimental phase for both Alpha and Theta bands. Considering that the interviewer conducted the interview repeatedly, each time interacting with new interviewees, an additional cluster‐level statistic (independent *t*‐test) was performed between the first and last five interviews undertaken in each group (DG and NDG) to account for the interviewer's habituation to the task. The present study addresses the problem of multiple comparisons, a major limitation in EEG studies [96], by correcting the results with the False Discovery Rate (FDR) correction [97−99] and using the nonparametric cluster‐based permutation test, which controls the family‐wise error rate within the EEG connectivity space [100]. The nonparametric cluster‐based approach clusters neighboring electrode pairs exhibiting similar effects and estimates statistical significance through resampling and permutation (*N* = 5000) [67]. Specifically, electrode pairs were clustered using an adjacency matrix based on the standard 10–20 EEG montage, which defines neighboring electrodes by their spatial proximity on the scalp [67, 75]. Only results from clustered electrode pairs that exceeded the cluster level threshold (*p* < 0.05) were interpreted using a visualization threshold > 3 (for both *t‐* and *F*‐values), as done in previous EEG hyperscanning studies [66, 67, 101−103]. Cohen's *d* was calculated to assess the effect sizes, according to previous EEG hyperscanning studies [66, 104, 105].

To assess differences in HR coherence, 3 × 2 repeated‐measures ANOVAs were conducted with experimental phase (First Direct Gaze vs. Interview vs. Second Direct Gaze) as within‐subjects factor and group (DG vs. NDG) as between‐subjects factor for both LF and HF bands, using JASP software (v. 0.18.3) [95]. Effect sizes were reported using partial eta square ($\eta_{p}^{2}$). For ANOVAs that showed a significant group effect, post‐hoc comparisons were conducted. Lastly, to assess differences in the number of interviewees’ heartbeats between the groups, independent sample *t*‐tests were conducted on the number of interviewees’ heartbeats between the DG versus NDG during the First Direct Gaze, the Interview, and the Second Direct Gaze phases. The results were corrected for multiple comparisons using the FDR method [97].

To evaluate associations between behavioral measures and indices of synchronization, point‐biserial correlations were conducted between the interviewer's accuracy in detecting deception (detected vs. not detected) and indices of both IBS and HR coherence. In addition, point‐biserial correlations were examined between the group assignment (DG vs. NDG) and indices of both IBS and HR coherence. Regarding IBS indices, only electrode pairs belonging to the significant clusters found in the independent *t*‐test in the two bands of interest (Theta during the First Direct Gaze and Alpha during the Interview) were included in the correlation analyses. As for HR coherence, correlation analyses were performed on both LF and HF bands for each experimental phase (First Direct Gaze, Interview, Second Direct Gaze). The correlation analyses described were conducted using JASP software (v. 0.18.3) [95]. The FDR correction method was used to correct *p‐*values for multiple comparisons [97].

## 3 Results

Independent *t*‐tests performed to evaluate the differences in age, education, motivation, and maladaptive personality traits between the DG and the NDG showed no significant differences (Table S3).

### 3.1 Interbrain Synchronization

**FIGURE 2:**
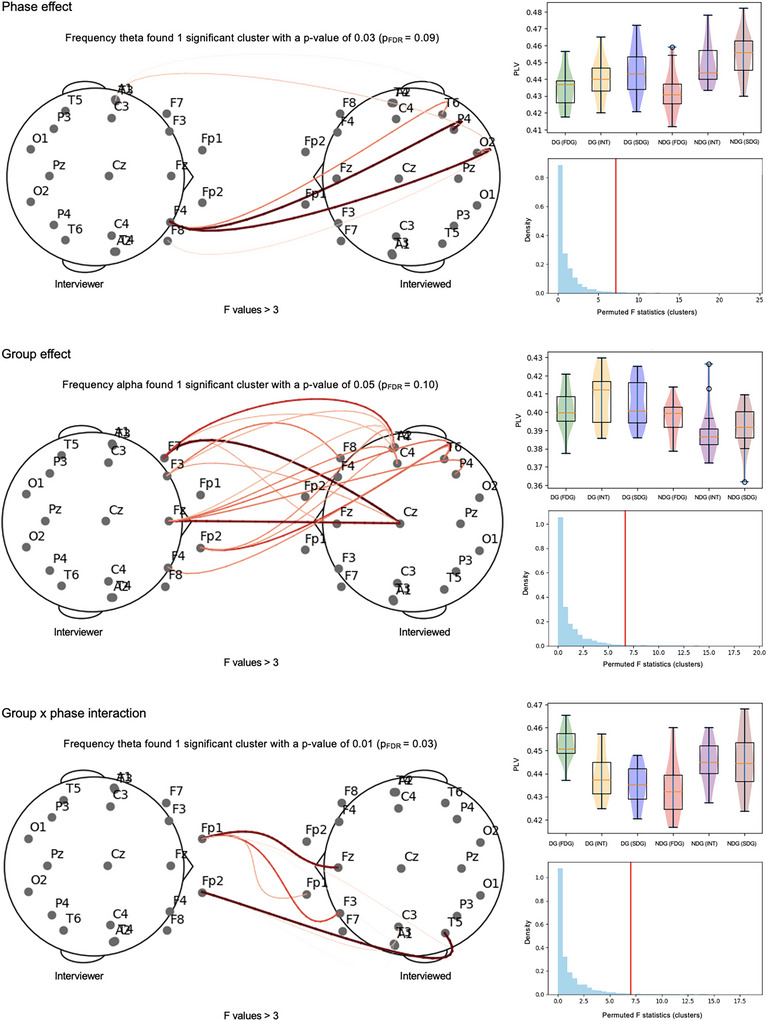
Graphical representation of the significant clusters found from the 3 (First Direct Gaze vs. Interview vs. Second Direct Gaze) × 2 (DG vs. NDG) repeated‐measures ANOVAs in the Theta and Alpha bands. Red lines represent *F*‐values (threshold > 3), with darker lines indicating greater differences in IBS. On the right, the violin plots of the distribution of the average PLV values evaluated on the electrode pairs belonging to the significant clusters and the cluster‐based permutation tests (*N* = 5000) with the red vertical line indicating the position of the cluster statistic relative to the distribution of the permuted data. Abbreviations: DG, deception group; FDG, first direct gaze; FDR, false‐discovery rate correction; INT, interview; NDG, non‐deception group; SDG, second direct gaze.

Cluster‐based 3 × 2 repeated‐measures ANOVAs showed a main effect of phase in a cluster in the Theta band, *F*_mean_ = 7.14 (*F*_min_ = 3.69, *F*_max_ = 11.36), *p* = 0.03, which included left fronto‐temporal connections (Figure 2, “Phase effect”). A main effect of group was found in a cluster in the Alpha band, *F*_mean_ = 6.65 (*F*_min_ = 4.68, *F*_max_ = 10.35), *p* = 0.05, which involved bilateral prefrontal and fronto‐medial connections, with increased IBS in the DG than in the NDG (Figure 2, “Group effect”). Lastly, a group × phase interaction effect was found in a cluster in the Theta band, *F*_mean_ = 6.99 (*F*_min_ = 3.49, *F*_max_ = 12.52) *p* = 0.01, which involved left fronto‐temporal connections (Figure 2, “Group × phase interaction”). After FDR correction for multiple comparisons, the group × phase interaction remained significant (*p*_FDR_ = 0.03), while the phase (*p*_FDR_ = 0.09) and the group (*p*_FDR_ = 0.10) effects did not survive the correction (see Supplementary Materials, Figure S1 for *F*‐values matrices of Theta band and Figure S2 for *F*‐values matrices of Alpha band).

The results of the 3 × 2 repeated‐measures ANOVAs conducted on the reshuffled dataset showed no significant effect on IBS in either the Theta band or the Alpha band, supporting the results of the real dyads.

**FIGURE 3:**
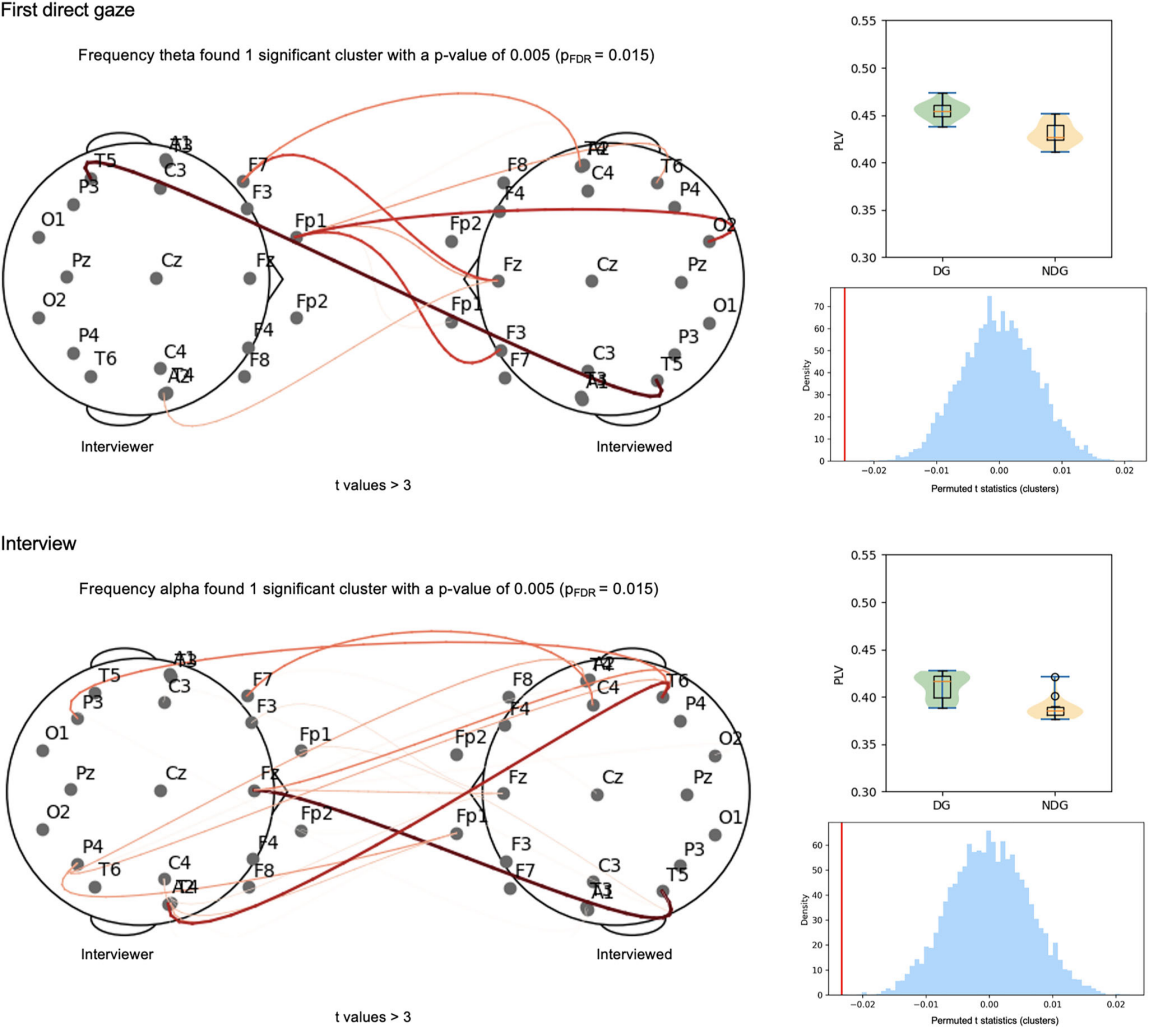
Graphical representation of significant clusters found from the independent‐samples *t*‐test between the deception group (DG) and the non‐deception group (NDG) in the Theta band during the First Direct Gaze and in the Alpha band during the Interview. Red lines represent positive *t*‐values (DG IBS > NDG IBS) (threshold > 3), with darker lines indicating a greater difference in the IBS between DG and NDG. On the right, the violin plots of the distribution of the average PLV values evaluated on the electrode pairs belonging to the significant clusters and the cluster‐based permutation tests (*N* = 5000) with the red vertical line indicating the position of the cluster statistic relative to the distribution of the permuted data. Note that FDR represents false‐discovery rate correction.

Cluster‐based independent *t*‐test analysis over the frequency bands of interest revealed a significant cluster in the Theta band, *t*_mean_ = 1.20 (*t*_min_ = 0.05, *t*_max_ = 4.01), *p* = 0.005, mainly involving left frontal electrodes of the interviewer and highlighting increased IBS for DG compared to NDG before the interview (Figure 3, “First Direct Gaze”). Moreover, a significant cluster in the Alpha band during the interview, *t*_mean_ = 1.66 (*t*_min_ = 0.06, *t*_max_ = 4.20), *p* = 0.005 (Figure 3, “Interview”), showed an increase of IBS for DG compared to NDG, involving mainly right temporo‐parietal electrodes of the interviewer. After FDR correction, both the clusters found in the Theta band (*p*_FDR_ = 0.015) and the Alpha band (*p*_FDR_ = 0.015) remained significant (see Supplementary Materials, Figure S3 for *t*‐values matrices of Theta band and Figure S4 for *t*‐values matrices of Alpha band). The greater differences between the IBSs of the two groups (darker lines) involved the left temporal electrode of the interviewees during both First Direct Gaze in the theta band and Interview in the alpha band. During the Second Direct Gaze, no significant clusters were found in either the Theta band (all *p* > 0.06; all *p*_FDR_ > 0.12) or the Alpha band (all *p* > 0.25; all *p*_FDR_ > 0.30) (Figures S3 and S4).

Regarding effect sizes, for the DG versus NDG comparisons in the First Direct Gaze, Cohen's *d* indicated a range between large and very large effect sizes (1.15 < Cohen's *d* < 1.55; mean = 1.31). Similarly, during the Interview, Cohen's *d* indicated a range between large and very large effect sizes (1.13 < Cohen's *d* < 1.58; mean = 1.25). The large and very large effect sizes found in both experimental phases support the findings that deception is associated with an increase in IBS both before and during the deceptive interaction.

Cluster‐based analyses (independent *t*‐test) conducted to assess possible differences in IBS between the first and last five interviews showed no significant cluster in both DG and NDG (see Supplementary Materials, Figures S5 and S6 for *t*‐values and *p*‐values).

### 3.2 Heart Rate Coherence and Heartbeat Results

The results of the repeated‐measures 3 (First Direct Gaze vs. Interview vs. Second Direct Gaze) × 2 (DG vs. NDG) ANOVA for HR coherence in the LF band showed a main effect of phase, *F*(2, 56) = 47.95, *p*<0.001, $\eta_{p}^{2}$ = 0.63. After FDR correction, the effect of phase for HR coherence in the LF band remained significant (*p*_FDR_ = 0.003). Neither the group main effect (*p* = 0.54; *p*_FDR_ = 0.68) nor the group × phase interaction (*p* = 0.57; *p*_FDR_ = 0.68) were significant. Regarding HR coherence in HF band, the ANOVA showed a main effect of phase, *F*(2, 56) = 33.55, *p*<0.001, $\eta_{p}^{2}$ = 0.55 and group, *F*(1, 28) = 7.02, *p* = 0.01, $\eta_{p}^{2}$ = 0.20, whereby the DG (0.68 ± 0.03) showed significantly lower HR coherence than the NDG (0.71 ± 0.03). After FDR correction, the effect of phase (*p*_FDR_ = 0.003) and group (*p*_FDR_ = 0.02) for HR coherence in the HF band remained significant. The group × phase interaction was not significant (*p* = 0.71; *p*_FDR_ = 0.71) (Table 1).

**TABLE 1:** The results of the repeated‐measures 3 (First Direct Gaze vs. Interview vs. Second Direct Gaze) × 2 (DG vs. NDG) ANOVAs for HR coherence in low and high frequency bands.

Frequency band	Effect	*F*	*p*‐value	*p_FDR_*	$\eta_{p}^{2}$
HR coherence in LF band	Phase	*F*(2, 56) = 47.95	< 0.001	0.003	0.63
	Group	*F*(1, 28) = 0.38	0.54	0.68	0.01
	Group × Phase	*F*(2, 56) = 0.57	0.57	0.68	0.02
HR coherence in HF band	Phase	*F*(2, 56) = 33.55	< 0.001	0.003	0.55
	Group	*F*(1, 28) = 7.02	0.01	0.02	0.20
	Group × Phase	*F*(2, 56) = 0.35	0.71	0.71	0.01

Abbreviations: FDR, false‐discovery rate correction; HF, high frequency; HR, heart rate; LF, low frequency.

The participants’ heartbeats of the DG, compared to those of the NDG, did not differ during the First Direct Gaze (M_DG_ = 159.0 ± 16.6, M_NDG_ = 153.1 ± 25.8; *t*(28) = 0.740, *p* = 0.465, *p*_FDR_ = 0.465), the Interview (M_DG_ = 270.1 ± 41.1, M_NDG_ = 236.5 ± 53.3; *t*(28) = 1.928, *p* = 0.064, *p*_FDR_ = 0.192), and the Second Direct Gaze (M_DG_ = 162.9 ± 23.3, M_NDG_ = 156.5 ± 21.0; *t*(28) = 0.791, *p* = 0.436, *p*_FDR_ = 0.465).

### 3.3 Associations Between Behavioral Measures and Synchrony Indices

The interviewer accurately identified deception in 67% of cases (10 out of 15 dyads) in the DG. No significant correlations were found between interviewer accuracy in detecting deception (detected vs. not detected) and IBS or HR coherence indices (all *p* and *p*_FDR_ > 0.05) (Table S4). Regarding correlations between the group assignment (DG vs. NDG) and IBS indices, several significant associations—all positive—were found in both Theta and Alpha bands. The strongest associations were found in the fronto‐temporal and fronto‐parietal connections (e.g., Fp1−F3, Fp1−T4, Fp1−O2, F7−Fz, F7−T4, and T5−T5 in the Theta band and Fz−T5 and P4−T6 in the Alpha band), indicating higher IBS in the DG than in the NDG. In all significant electrode pairs, *r_pb_* values ranged from 0.49 to 0.62 (all *p*‐values < 0.005; all *p*_FDR_ < 0.006). Regarding the correlations between the group assignment (DG vs. NDG) and HR coherence indices, a negative significant association was found only during the Interview phase in the HF band (*r_pb_* = −0.46, *p*_FDR_ = 0.012), indicating lower HR coherence in the DG than in NDG (Table S5).

## 4 Discussion

The present study aimed to evaluate the differences in IBS and HR synchrony (interviewer−interviewee) between a group in which the interviewee deceived (deception group, “DG”) and a group in which the interviewee did not deceive (non‐deception group, “NDG”). The main results showed an effect of the group (DG vs. NDG) on IBS in an Alpha band cluster and both an effect of phase (First Direct Gaze vs. Interview vs. Second Direct Gaze) and a group × phase interaction on IBS in Theta band clusters, although only the interaction survived the FDR correction. Regarding HR coherence, the study showed an effect of phase on both the low and high frequency bands and an effect of group on the high frequency band. Specifically, DG exhibited higher IBS before the interview in a theta band (4−7 Hz) cluster and during the interview in an alpha band (8−12 Hz) cluster, while displaying decreased HR coherence in the high frequency band compared to NDG. Lastly, correlations with behavioral measures showed no significant associations of the interviewer's accuracy in detecting deception with neural and autonomic synchronization in DG, while revealing positive associations of deception with IBS indices and negative associations with HR coherence indices.

These results show that in dyads where one individual deceives, and the other tries to detect the deception, their neural activations would be more synchronized and their heart rate less coordinated than in dyads where the individual tells the truth. The higher IBS observed in the DG compared to the NDG is consistent with previous literature showing an increase in IBS during deceptive acts compared to honest ones [4]. Lying and its detection seem to foster in both interlocutors a tendency to evaluate socioemotional cues to analyze their behavior, for example, looking into each other's eyes is a relevant cue for speculating on the internal states of others [36, 37, 38, 106, 107]. Accordingly, research on the neural basis of deception showed activation of areas associated with mentalization, socioemotional processing, and cognitive control [4, 15, 16]. On the one hand, the present study's findings suggest that these neural processes could be more synchronized in dyads where one individual lies, compared to dyads where one individual tells the truth [4]. On the other hand, the results suggest that autonomic activations (heart rate) could follow different activation patterns within the dyad in which an individual has a deceptive intent. Specifically, the decreased heart rate coherence in the deceptive group emerged in the high frequency band, suggesting lower synchronization at the parasympathetic level [108, 109]. In this regard, previous scientific research showed that increased synchronization of heart rate was associated with greater dyadic trust [59] and involvement in cooperative tasks in both high and low frequency bands [92], while lower congruence of ECG signals was found in competitive contexts [60]. Several studies highlighted that when individuals are deceptive, they experience increased autonomic activity, which could reflect a higher physiological arousal and cognitive load in generating deceptive information [29]. Moreover, autonomic activations seem to be associated with involuntary bodily responses and emotional involvement, while neural activations are associated with high‐level cognitive and affective regulation [110]. Accordingly, it could be hypothesized that shared attention and processing of social‐emotional states during deceptive acts would be associated with increased IBS, while processes less subject to cognitive control would be associated with lower autonomic synchrony.

In addition, it is worth noting that the heartbeats of DG interviewees did not differ significantly from those of NDG, suggesting that simultaneous, rather than just intraindividual, assessment of autonomous activities would be an interesting indicator for identifying physiological correlates of deception. Moreover, although previous research showed an increase in heart rate during deceptive behaviors [29, 30, 31], the results of the present study suggest that deception may underlie regulatory mechanisms beyond just autonomic arousal. In socially interactive contexts, such as the experimental paradigm used in the study, deceivers must maintain credibility, appear natural, and sustain a cooperative conversational tone [8, 36]. These activities could potentially foster greater regulation, allowing the deceiver to suppress physiological arousal and appear calm and convincing.

In the present study, during deception (Interview phase), higher IBS in the DG compared to the NDG was specifically found in alpha band activity. It has been suggested that alpha activity has a potential role in maintaining attention to the reactions of others [55]. Moreover, previous studies found an increase in alpha band IBS during motor coordination tasks [111] and flight phases involving high cooperativity [40, 65]. Interestingly, the results of the present study suggest that this increase in IBS may not be related to greater collaboration or coordination per se, but rather to a greater shared attention to the task. Indeed, it was proposed that alpha activity would be related to understanding the mental states, emotions, and behavior of others [112]. Thus, the enhanced alpha IBS between individuals during mental coordination might also reflect enhanced neural couplings of the brain activity associated with sharing/understanding others’ mental states to coordinate with others mentally [112]. It is possible to hypothesize that lying processes involve attentional control and attempts to understand others, which are more shared in dyads in which one individual lies than in dyads in which one individual does not lie, emphasizing the high intersubjectivity involved in this process.

Interestingly, the present study showed that the theta band IBS increased before the interview (First Direct Gaze phase). In this regard, several studies highlighted the role of theta rhythm in social and emotional processes [113]. It has been proposed that theta activity is associated with empathy processes [114], where the increased IBS might reflect a social understanding among interacting individuals [115]. Consistently, it could be argued that even the intention to deceive another person, without effectively performing the deceptive act, is associated with increased IBS due to the deceiver's efforts to comprehend the interlocutor's reactions. The findings of the present study concerning large and very large effect sizes of increased IBS in the deception group both before and during the interview support the results that neural synchronization processes would already be occurring in the preparatory phase of lying and maintained during the deceptive verbal interaction with another person.

Furthermore, it is worth noting that the greater IBS in DG specifically involved the left temporal electrode of the interviewee during both the First Direct Gaze phase in the theta band and the Interview phase in the alpha band. This finding suggests that the left temporal region could play a relevant role not only in deceptive behavior, as highlighted by previous studies [26, 116], but also in deception planning, considering the reactions and behaviors of the person being deceived.

Finally, the present study shed light on the cognitive dimension related to synchronization during deceptive interactions. Specifically, the absence of significant correlations between the interviewer's accuracy in detecting deception with IBS and HR coherence suggests that this neural and autonomic coupling is not the correlate of explicit awareness of deception detection. Differently, the deception group was positively associated with IBS and negatively associated with HR coherence. These results support the hypothesis that the synchronization found could be the correlate of the manipulative processes by the deceiver.

While the present study offers innovative insights into the neurophysiological processes underlying deception, some limitations should be highlighted. First, despite the sample size being similar to [52, 117] or higher [64, 65] than previous hyperscanning studies, the small number of participants could have weakened the statistical power of the present study. In addition, although the direct gaze phases allowed for the investigation of the neural and physiological synchronization dynamics during the entire deceptive interaction, including the period before and after the deceptive act itself, these might have introduced several confounding factors that could not be directly controlled, such as increased arousal and gaze avoidance by the deceivers that the interviewer might have recognized. Another relevant limitation of the study concerns the absence of a resting‐state baseline assessment, which did not allow the evaluation of interindividual differences in both IBS and HR coherence measures. Future research investigating synchronization during deception should include a resting‐state recording before the beginning of the experimental condition to control for baseline levels of synchronization, thus allowing more accurate estimation of task‐related effects. Lastly, it is necessary to consider that the number of EEG electrodes was limited, and therefore, the directionality of the neural signals assessed with the hyperscanning technique could not be determined. Future research should strengthen the results of the present study by replicating them on a larger sample and using a higher number of electrodes. Furthermore, future studies could develop and standardize experimental paradigms to investigate the interpersonal neurobiological basis of deception. Additionally, these studies should include an experimental procedure that measures a baseline of IBS and resting heart rate before any tasks to control for intraindividual variability.

## 5 Conclusion

In conclusion, the present study provided new insight into the interpersonal neural and autonomic basis of deception. The results showed that DG exhibited greater synchrony at the neural level while displaying lower heart rate synchrony compared to NDG. This discrepancy between neural and autonomic synchrony suggests that deception is a complex process involving cognitive coordination between interlocutors, but may lead to differences in their emotional and autonomic states. In addition, the results of the present study suggest that the left temporal region might play a relevant role in deceptive planning and behavior. Overall, the results of the present study highlight the importance of adopting an interpersonal perspective to investigate deception. Rather than being a solely individual act, deception emerges within a dynamic exchange between deceiver and deceived. By framing deception as a relational rather than exclusively individual phenomenon, the present study opens further applications in the forensic and clinical fields. Indeed, these findings could have implications for the development of advanced lie‐detection techniques. In particular, simultaneous intercerebral and heartbeat synchronization analyses could be integrated with other methodologies, such as polygraphs, to better understand the behavior and physiological reactions involved in dyadic interaction during interviews or interrogations to assess deception. This could be useful for interpreting behavioral cues during investigative interviews and providing insights to enhance interview management techniques, thereby helping investigators detect signs of discomfort and tension associated with deception within the interactive exchange between interviewer and interviewee.

## Author Contributions

Conceptualization: G.V. and C.L. Data curation: G.V. and F.L. Formal analysis: G.V. and C.L. Funding acquisition: G.V. and C.L. Investigation: G.V. and F.L. Methodology: G.V., C.L., and E.G. Project administration: G.V., C.L., and F.L. Resources: G.V. and V.C. Software: G.V. Supervision: C.L. Validation: G.V., C.L., F.L., E.G., and V.C. Visualization: G.V., C.L., F.L., E.G., and V.C. Writing – original draft: G.V., C.L., F.L., E.G., and V.C. Writing – review and editing: G.V., C.L., F.L., and E.G.

## Funding

The study received funding from the PON R&I (research and innovation) program 2014–2020 under a grant agreement by the Italian Ministry of University and Research (MUR), 10612021, and from “Progetti per Avvio alla Ricerca—Tipo 1”, Sapienza University of Rome, protocol number: AR1221816C5BCD51.

## Conflicts of Interest

The authors declare that they have no known competing financial interests or personal relationships that could have appeared to influence the work reported in this paper.

## Ethics Approval Statement

The present study was approved by the Ethics Committee of the Department of Dynamic and Clinical Psychology, and Health Studies, Sapienza University (protocol number: 0000589).

## Supporting information




**Supplementary Material**: nyas70217‐sup‐0001‐SuppMat.docx

## Data Availability

The data are available upon request from the corresponding author. The data are not publicly available for privacy reasons: questionnaires and recordings contain information that could compromise participants’ privacy. The EEG analysis codes are publicly available: DOI 10.17605/OSF.IO/D2U3C.
