# Supplementary material for: Deception Affects Interbrain Electroencephalographic and Autonomic Synchronization Within a Dyad: A Hyperscanning Study
Source: Ann N Y Acad Sci. 2026 Feb 11;1556(1):e70217. doi: 10.1111/nyas.70217 (PMC12894808; doi:10.1111/nyas.70217)
Supplement: Supplementary file 1 — Supplementary Material: nyas70217‐sup‐0001‐SuppMat.docx [file NYAS-1556-0-s001.docx]

**Supplementary materials**

**Table S1**. Structured interview.

|  | Questions evaluating the presence of non-restricted objects | Questions evaluating the presence of prohibited objects | Unexpected questions about general information |
| --- | --- | --- | --- |
| 1 | Does your backpack contain medication? | Does your backpack contain prohibited items? | Are you currently a student? |
| 2 | Has your backpack been filled by you? | Does your backpack contain liquids containing alcohol? | Do you feel comfortable? |
| 3 | Does your backpack contain stationery? | Are any of the items in your backpack allowed in the department? | Are you upset? |
| 4 | Does your backpack contain computer equipment? | Does your backpack contain flammable liquids? | Do you think there is traffic today? |
| 5 | Does your backpack contain clothes? | Are you sure there are no prohibited items in your backpack? | Will he/she stay in the Department? |
| 6 | Does your backpack contain sunglasses? | Does your backpack contain sharp objects? | Do you think there will be good weather today? |
| 7 | Does your backpack contain notebooks? | Does your backpack contain objects that could be used to harm anyone? | Did you have a good morning? |
| 8 | Does your backpack contain books? | Does your backpack contain tools or other dangerous objects? | Are you feeling well? |
| 9 | Does your backpack contain liquids? | Does your backpack contain illegal substances in the Department? | Do you feel cold at the moment? |
| 10 | Does your backpack contain electronic items? | Does your backpack contain sharp objects? | Is today your birthday? |

**Table S2**. Means of rejected epochs of DG and NDG related to the 3 phases of the experimental procedure.

| Experimental procedure phases | Mean of rejected epochs | | |
| --- | --- | --- | --- |
|  | DG | NDG | Total |
| First Direct Gaze (128s) | 20.6% | 23.6%  29.1%  19.2% | 22.1% |
| Interview (192s) | 22.7% |  | 25.9% |
| Second Direct Gaze (128s) | 15.7% |  | 17.5% |

*Note*. “DG” = Deception Group; “NDG” = Non-Deception Group.

**Table S3**. Differences between the Deception Group and Non-Deception Group on demographics and personality dimensions. Mean, standard deviations (*SD*), and independent t-tests of age, years of education, motivation, and the Personality Inventory for DSM-5 - Brief Form (PID-5-BF), between the Deception and the Non-Deception groups.

| Variables | Deception Group  (N = 15) | | Non-Deception Group  (N = 15) | | Independent  t-test | | |
| --- | --- | --- | --- | --- | --- | --- | --- |
|  | Mean | *SD* | Mean | *SD* | *t* | df | *p*-value |
| Age | 26.20 | 3.95 | 24.13 | 3.14 | 1.59 | 28 | 0.12 |
| Education (years) | 16.00 | 1.46 | 15.47 | 1.68 | 0.93 | 28 | 0.36 |
| Motivation | 5.73 | 1.28 | 5.87 | 1.19 | -0.30 | 28 | 0.77 |
| Negative Affect | 1.37 | 0.58 | 1.09 | 0.54 | 1.36 | 28 | 0.19 |
| Detachment | 0.81 | 0.61 | 0.62 | 0.54 | 0.88 | 28 | 0.38 |
| Antagonism | 0.67 | 0.46 | 0.63 | 0.35 | 0.27 | 28 | 0.79 |
| Disinhibition | 0.81 | 0.58 | 0.76 | 0.61 | 0.24 | 28 | 0.81 |
| Psychoticism | 0.92 | 0.55 | 0.93 | 0.84 | -0.05 | 28 | 0.96 |
| PID-5-BF total score | 0.92 | 0.41 | 0.81 | 0.44 | 0.70 | 28 | 0.49 |

**Table S4**. Point-biserial correlations (*r_pb_*) and related p-values between the interviewer's accuracy in detecting deception (non-detected *vs*. detected) and the indices of inter-brain synchronization and heart rate coherence in the Deception Group.

| Synchrony indices | Non-detected (0) *vs*. Detected (1) |
| --- | --- |
| Theta Fp1-Fp1 FDG | -0.28 (*p* = 0.312, *p*_FDR_ = 0.831) |
| Theta Fp1-F3 FDG | 0.07 (*p* = 0.809, *p*_FDR_ = 0.884) |
| Theta Fp1-Fz FDG | -0.03 (*p* = 0.917, *p*_FDR_ = 0.968) |
| Theta Fp1-T4 FDG | 0.25 (*p* = 0.359, *p*_FDR_ = 0.831) |
| Theta Fp1-T6 FDG | -0.18 (*p* = 0.523, *p*_FDR_ = 0.831) |
| Theta Fp1-O2 FDG | -0.01 (*p* = 0.959, *p*_FDR_ = 0.969) |
| Theta F7-Fz FDG | 0.14 (*p* = 0.610, *p*_FDR_ = 0.831) |
| Theta F7-T4 FDG | 0.08 (*p* = 0.785, *p*_FDR_ = 0.884) |
| Theta T5-T5 FDG | -0.42 (*p* = 0.119, *p*_FDR_ = 0.831) |
| Theta A2-Fz FDG | 0.15 (*p* = 0.601, *p*_FDR_ = 0.831) |
| Alpha Fp1-T5 INT | -0.21 (*p* = 0.443, *p*_FDR_ = 0.831) |
| Alpha Fp2-T6 INT | -0.23 (*p* = 0.417, *p*_FDR_ = 0.831) |
| Alpha F7-Cz INT | -0.33 (*p* = 0.225, *p*_FDR_ = 0.831) |
| Alpha F7-C4 INT | -0.08 (*p* = 0.788, *p*_FDR_ = 0.884) |
| Alpha F3-Fz INT | -0.45 (*p* = 0.091, *p*_FDR_ = 0.831) |
| Alpha Fz-Fz INT | -0.41 (*p* = 0.132, *p*_FDR_ = 0.831) |
| Alpha Fz-T5 INT | -0.37 (*p* = 0.175, *p*_FDR_ = 0.831) |
| Alpha Fz-T6 INT | -0.41 (*p* = 0.125, *p*_FDR_ = 0.831) |
| Alpha Fz-O2 INT | -0.19 (*p* = 0.508, *p*_FDR_ = 0.831) |
| Alpha C3-T5 INT | 0.07 (*p* = 0.814, *p*_FDR_ = 0.884) |
| Alpha C4-Fp1 INT | -0.20 (*p* = 0.480, *p*_FDR_ = 0.831) |
| Alpha C4-Fz INT | -0.28 (*p* = 0.304, *p*_FDR_ = 0.831) |
| Alpha C4-T3 INT | -0.24 (*p* = 0.388, *p*_FDR_ = 0.831) |
| Alpha T4-T6 INT | 0.16 (*p* = 0.575, *p*_FDR_ = 0.831) |
| Alpha P3-T6 INT | 0.10 (*p* = 0.713, *p*_FDR_ = 0.884) |
| Alpha P3-A1 INT | -0.24 (*p* = 0.382, *p*_FDR_ = 0.831) |
| Alpha P4-Fp1 INT | -0.15 (*p* = 0.584, *p*_FDR_ = 0.831) |
| Alpha P4-C4 INT | 0.18 (*p* = 0.523, *p*_FDR_ = 0.831) |
| Alpha P4-T4 INT | -0.32 (*p* = 0.248, *p*_FDR_ = 0.831) |
| Alpha P4-T6 INT | -0.25 (*p* = 0.365, *p*_FDR_ = 0.831) |
| Alpha A2-Fp1 INT | -0.07 (*p* = 0.802, *p*_FDR_ = 0.884) |
| Alpha A2-T6 INT | -0.41 (*p* = 0.127, *p*_FDR_ = 0.831) |
| HR coherence LF FDG | 0.01 (*p* = 0.969, *p*_FDR_ = 0.969) |
| HR coherence HF FDG | -0.14 (*p* = 0.612, *p*_FDR_ = 0.831) |
| HR coherence LF INT | -0.17 (*p* = 0.552, *p*_FDR_ = 0.831) |
| HR coherence HF INT | -0.26 (*p* = 0.354, *p*_FDR_ = 0.831) |
| HR coherence LF SDG | -0.46 (*p* = 0.082, *p*_FDR_ = 0.831) |
| HR coherence HF SDG | 0.09 (*p* = 0.754, *p*_FDR_ = 0.884) |

*Note*. “HR” = Heart rate; “LF” = Low frequency; “HF” = High frequency; “FDG” = First Direct Gaze; “INT” = Interview; “SDG” = Second Direct Gaze; “FDR” = False Discovery Rate correction.

**Table S5**. Point-biserial correlations (*r_pb_*) and p-values between the group (Non-Deception Group *vs*. Deception Group) and the indices of inter-brain synchronization and heart rate coherence.

| Synchrony indices | NDG (0) *vs*. DG (1) |
| --- | --- |
| Theta Fp1-Fp1 FDG | 0.50 (*p* = 0.005, *p*_FDR_ = 0.006) |
| Theta Fp1-F3 FDG | 0.57 (*p* = 0.001, *p*_FDR_ = 0.004) |
| Theta Fp1-Fz FDG | 0.54 (*p* = 0.002, *p*_FDR_ = 0.004) |
| Theta Fp1-T4 FDG | 0.50 (*p* = 0.005, *p*_FDR_ = 0.006) |
| Theta Fp1-T6 FDG | 0.54 (*p* = 0.002, *p*_FDR_ = 0.004) |
| Theta Fp1-O2 FDG | 0.58 (*p* = 0.001, *p*_FDR_ = 0.004) |
| Theta F7-Fz FDG | 0.57 (*p* = 0.001, *p*_FDR_ = 0.004) |
| Theta F7-T4 FDG | 0.55 (*p* = 0.002, *p*_FDR_ = 0.004) |
| Theta T5-T5 FDG | 0.60 (*p* = 0.000, *p*_FDR_ = 0.000) |
| Theta A2-Fz FDG | 0.53 (*p* = 0.003, *p*_FDR_ = 0.006) |
| Alpha Fp1-T5 INT | 0.52 (*p* = 0.003, *p*_FDR_ = 0.006) |
| Alpha Fp2-T6 INT | 0.50 (*p* = 0.005, *p*_FDR_ = 0.006) |
| Alpha F7-Cz INT | 0.50 (*p* = 0.005, *p*_FDR_ = 0.006) |
| Alpha F7-C4 INT | 0.56 (*p* = 0.001, *p*_FDR_ = 0.004) |
| Alpha F3-Fz INT | 0.51 (*p* = 0.004, *p*_FDR_ = 0.006) |
| Alpha Fz-Fz INT | 0.53 (*p* = 0.002, *p*_FDR_ = 0.004) |
| Alpha Fz-T5 INT | 0.62 (*p* = 0.000, *p*_FDR_ = 0.000) |
| Alpha Fz-T6 INT | 0.56 (*p* = 0.001, *p*_FDR_ = 0.004) |
| Alpha Fz-O2 INT | 0.50 (*p* = 0.005, *p*_FDR_ = 0.006) |
| Alpha C3-T5 INT | 0.50 (*p* = 0.005, *p*_FDR_ = 0.006) |
| Alpha C4-Fp1 INT | 0.53 (*p* = 0.003, *p*_FDR_ = 0.006) |
| Alpha C4-Fz INT | 0.51 (*p* = 0.004, *p*_FDR_ = 0.006) |
| Alpha C4-T3 INT | 0.49 (*p* = 0.005, *p*_FDR_ = 0.006) |
| Alpha T4-T6 INT | 0.54 (*p* = 0.002, *p*_FDR_ = 0.004) |
| Alpha P3-T6 INT | 0.56 (*p* = 0.001, *p*_FDR_ = 0.004) |
| Alpha P3-A1 INT | 0.49 (*p* = 0.005, *p*_FDR_ = 0.006) |
| Alpha P4-Fp1 INT | 0.55 (*p* = 0.002, *p*_FDR_ = 0.004) |
| Alpha P4-C4 INT | 0.50 (*p* = 0.005, *p*_FDR_ = 0.006) |
| Alpha P4-T4 INT | 0.54 (*p* = 0.002, *p*_FDR_ = 0.004) |
| Alpha P4-T6 INT | 0.54 (*p* = 0.002, *p*_FDR_ = 0.004) |
| Alpha A2-Fp1 INT | 0.50 (*p* = 0.005, *p*_FDR_ = 0.006) |
| Alpha A2-T6 INT | 0.60 (*p* = 0.000, *p*_FDR_ = 0.000) |
| HR coherence LF FDG | 0.06 (*p* = 0.737, *p*_FDR_ = 0.757) |
| HR coherence HF FDG | -0.16 (*p* = 0.400, *p*_FDR_ = 0.422) |
| HR coherence LF INT | 0.24 (*p* = 0.208, *p*_FDR_ = 0.226) |
| HR coherence HF INT | -0.46 (*p* = 0.010, *p*_FDR_ = 0.012) |
| HR coherence LF SDG | -0.02 (*p* = 0.918, *p*_FDR_ = 0.918) |
| HR coherence HF SDG | -0.30 (*p* = 0.104, *p*_FDR_ = 0.116) |

*Note*. “HR” = Heart rate; “LF” = Low frequency; “HF” = High frequency; “FDG” = First Direct Gaze; “INT” = Interview; “SDG” = Second Direct Gaze; “FDR” = False Discovery Rate correction.

**Figure S1**. *F*-values matrices of the phase effect (A), group effect (B), and phase × group interaction (C) of the 3 × 2 repeated-measures ANOVA conducted for inter-brain synchronization on the Theta band.

**
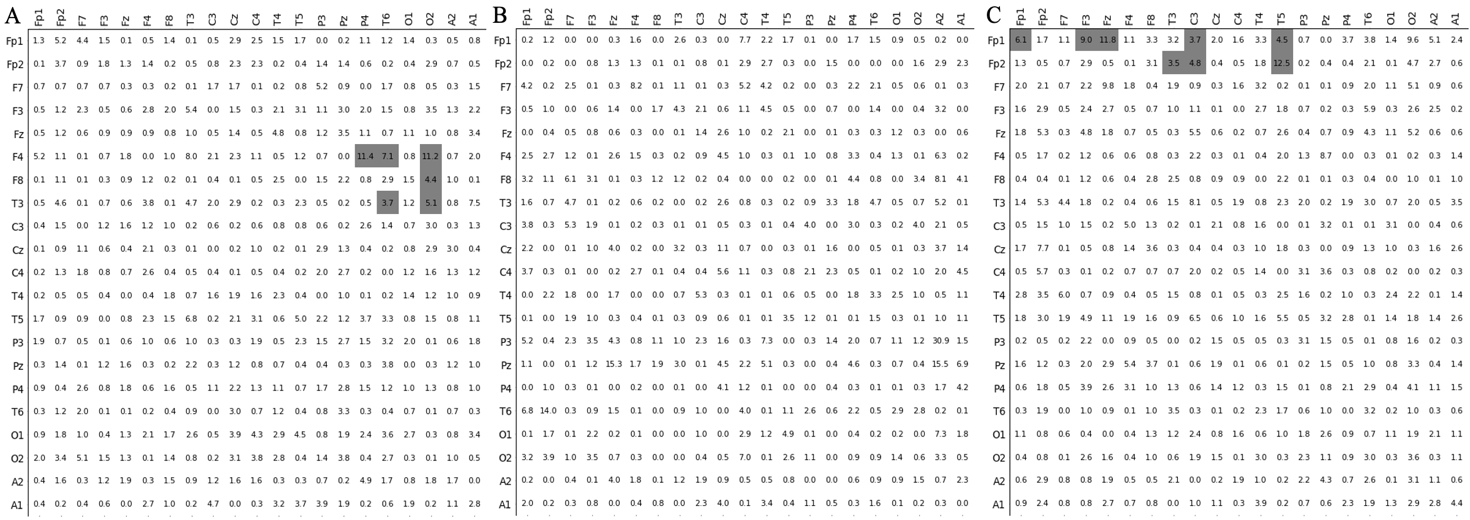
**

*Note*. In gray are shown the electrode pairs belonging to the significant clusters found (*F* > 3), respectively, for phase effect (A), *F*_mean_=7.14 *(F*_min_=3.69, *F*_max_=11.36), *p*=0.03, *p*_FDR_=0.09, and for group × phase interaction (C), *F*_mean_=6.99 *(F*_min_=3.49, *F*_max_=12.52) *p*=0.01, *p*_FDR_=0.03. For group effect (B) the clusters found were not significant (all *p* > 0.11; all *p*_FDR_ > 0.16).

**Figure S2**. *F*-values matrices of the phase effect (A), group effect (B), and phase × group interaction (C) of the 3 × 2 repeated-measures ANOVA conducted for inter-brain synchronization on the Alpha band.

**
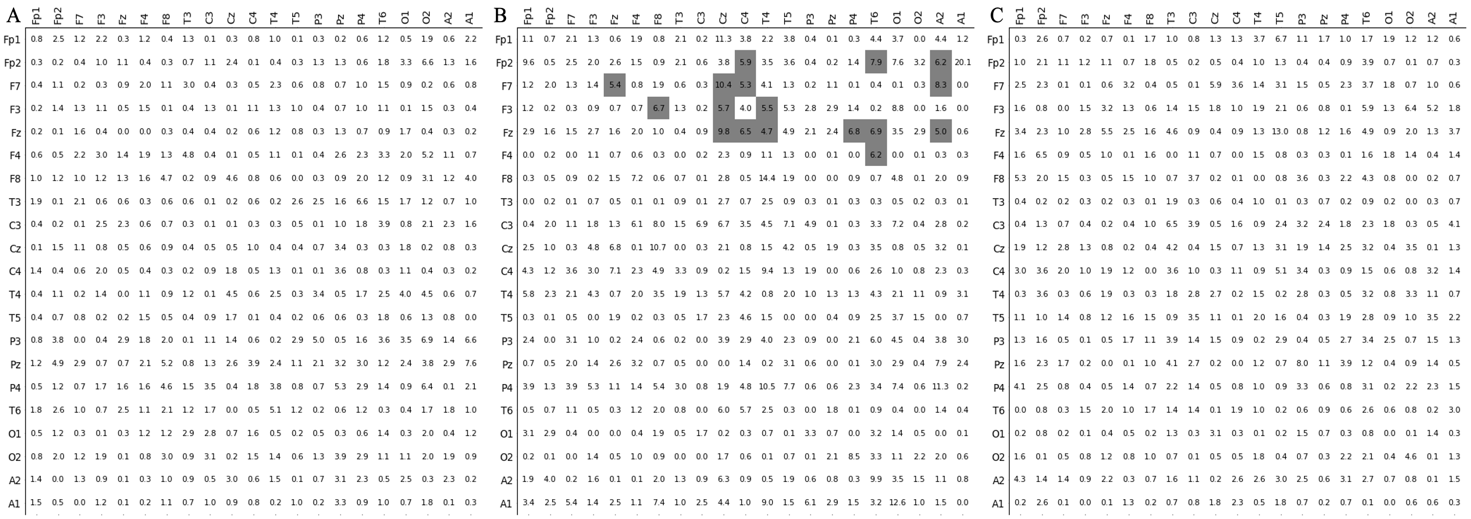
**

*Note*. In gray are shown the electrode pairs belonging to the significant cluster found (*F* > 3) for group effect (B), *F*_mean_= 6.65 (*F*_min_=4.68, *F*_max_=10.35), *p*=0.05, *p*_FDR_=0.10. For phase effect (A) (all *p* > 0.21; all *p*_FDR_ > 0.21) and for group × phase interaction (C) (all *p* > 0.13; all *p*_FDR_ > 0.16) the clusters found were not significant.

**Figure S3**. Matrices of *t*-values of the inter-brain synchronization differences between the Deception group (DG) and the Non-Deception group (NDG) during the First Direct Gaze (A), the Interview (B), and the Second Direct Gaze (C) on the Theta band.


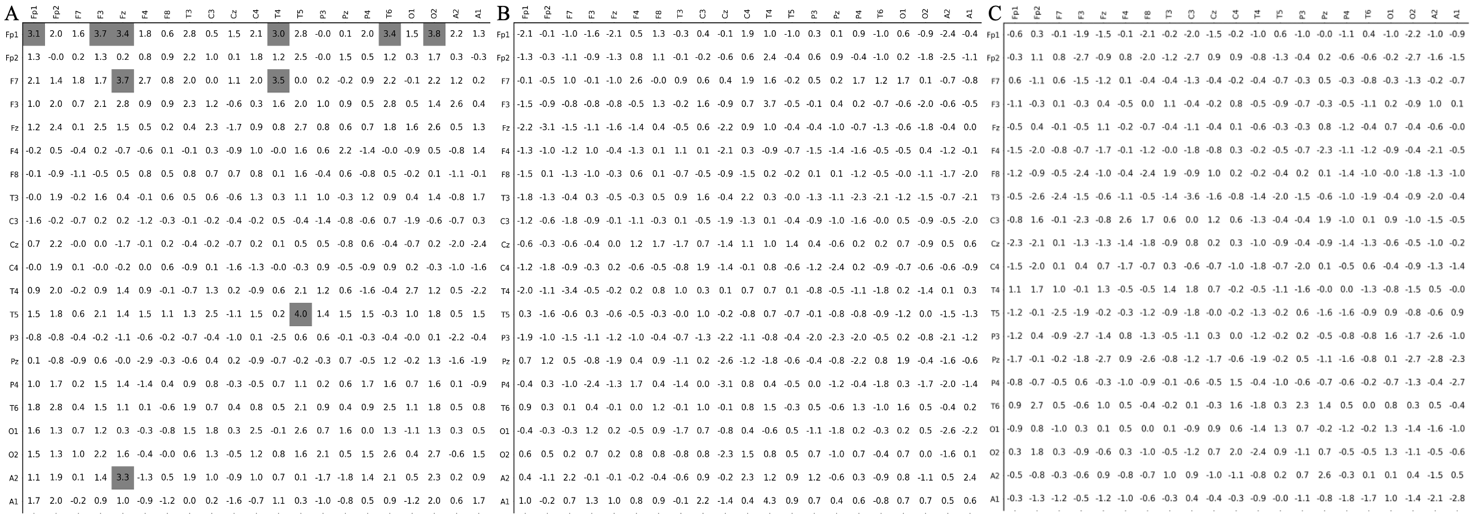


*Note*. In gray are shown the electrode pairs belonging to the significant cluster found *(t >* 3) during the First Direct Gaze (A), *t*_mean_=1.20*(t*_min_=0.05, *t*_max_=4.01), *p*=0.005, *p*_FDR_=0.015. During the Interview (B) (all *p* > 0.13, all *p*_FDR_ > 0.20) and during the Second Direct Gaze (C) (all *p* > 0.06, all *p*_FDR_ > 0.12) the clusters found were not significant.

**Figure S4**. Matrices of *t*-values of the inter-brain synchronization differences between the Deception group (DG) and the Non-Deception group (NDG) during the First Direct Gaze (A), the Interview (B), and the Second Direct Gaze (C) on the Alpha band.

**
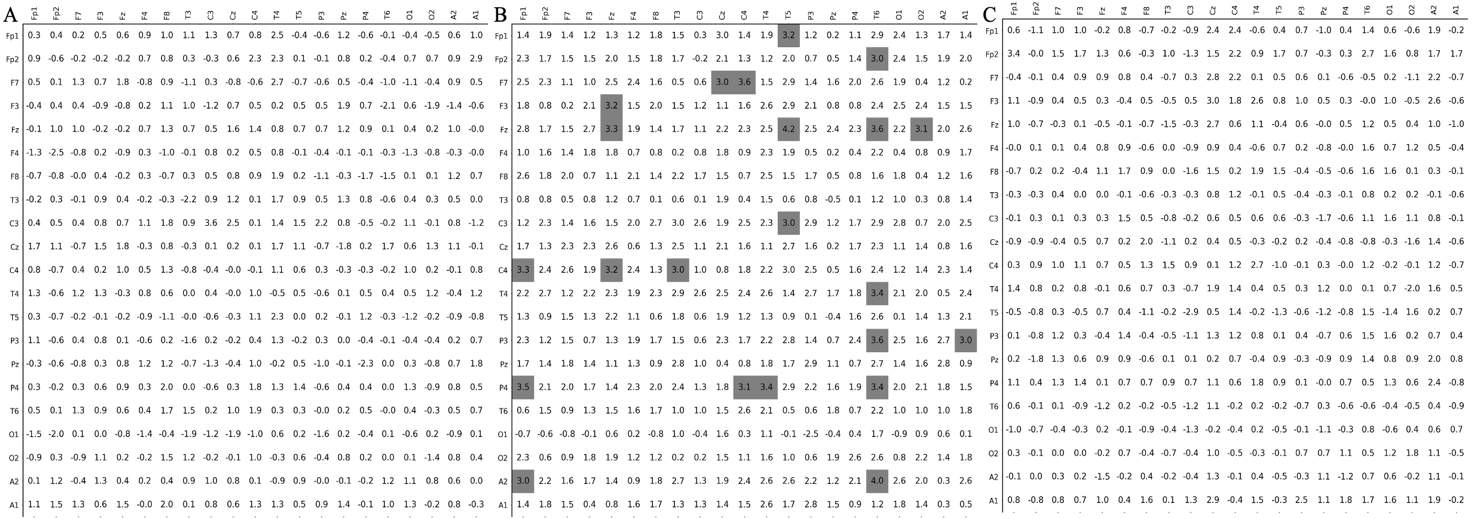
***Note*. In gray are shown the electrode pairs belonging to the significant cluster found *(t >* 3) during the Interview (B), *t*_mean_=1.66*(t*_min_=0.06, *t*_max_=4.20), *p*=0.005, *p*_FDR_=0.015. During the First Direct Gaze (A) (all *p* > 0.30, all *p*_FDR_ > 0.30) and during the Second Direct Gaze (C) (all *p* > 0.25, all *p*_FDR_ > 0.30) the clusters found were not significant.

**Figure S5.** Matrices of *t*-values of the inter-brain synchronization differences between the first five interviews and the last five interviews of the Deception Group (DG) on the Theta (A) and Alpha (B) bands.

**
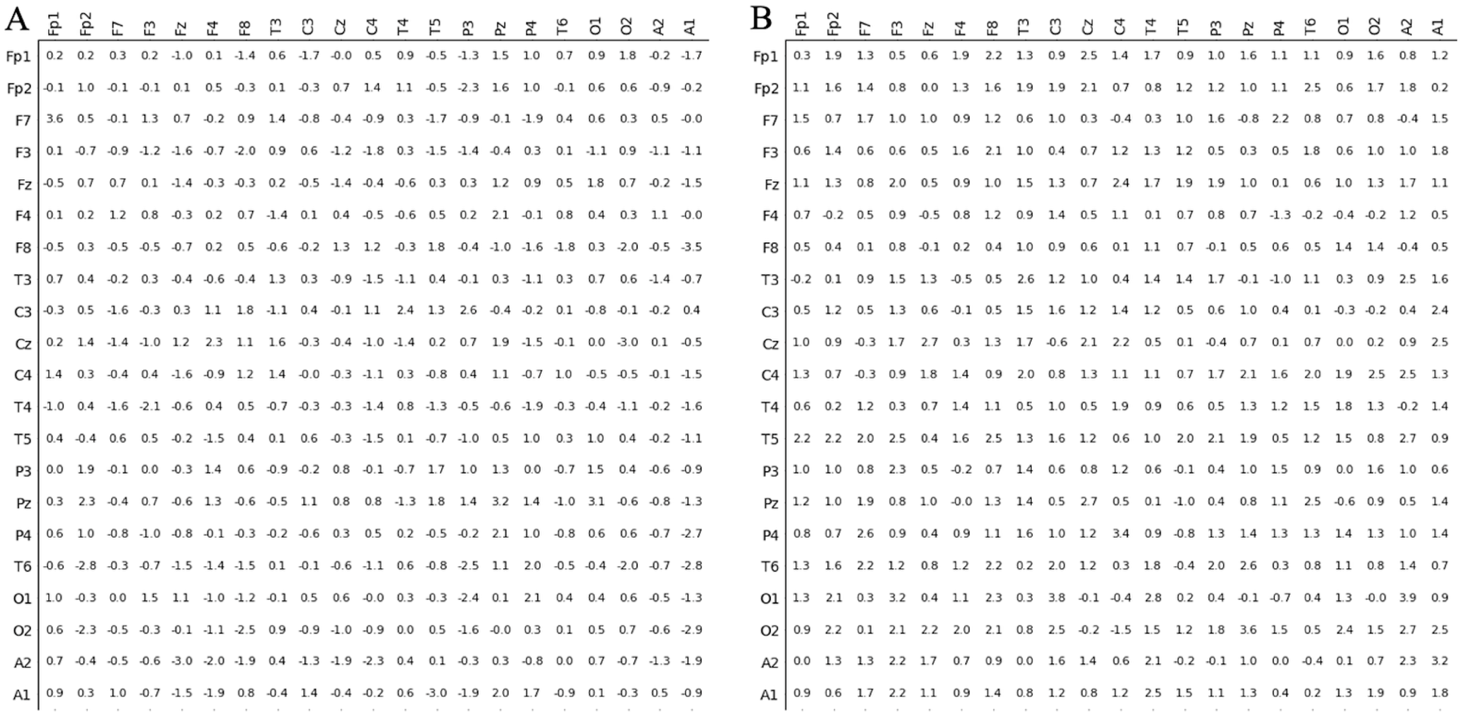
**

*Note*. The clusters found were not significant for either Theta band (all *p* > 0.38; all *p*_FDR_ > 0.38) or Alpha band (all *p* > 0.06; all *p*_FDR_ > 0.24).

**Figure S6.** Matrices of *t*-values of the inter-brain synchronization differences between the first five interviews and the last five interviews of the Non-Deception Group (NDG) on the Theta (A) and Alpha (B) bands.


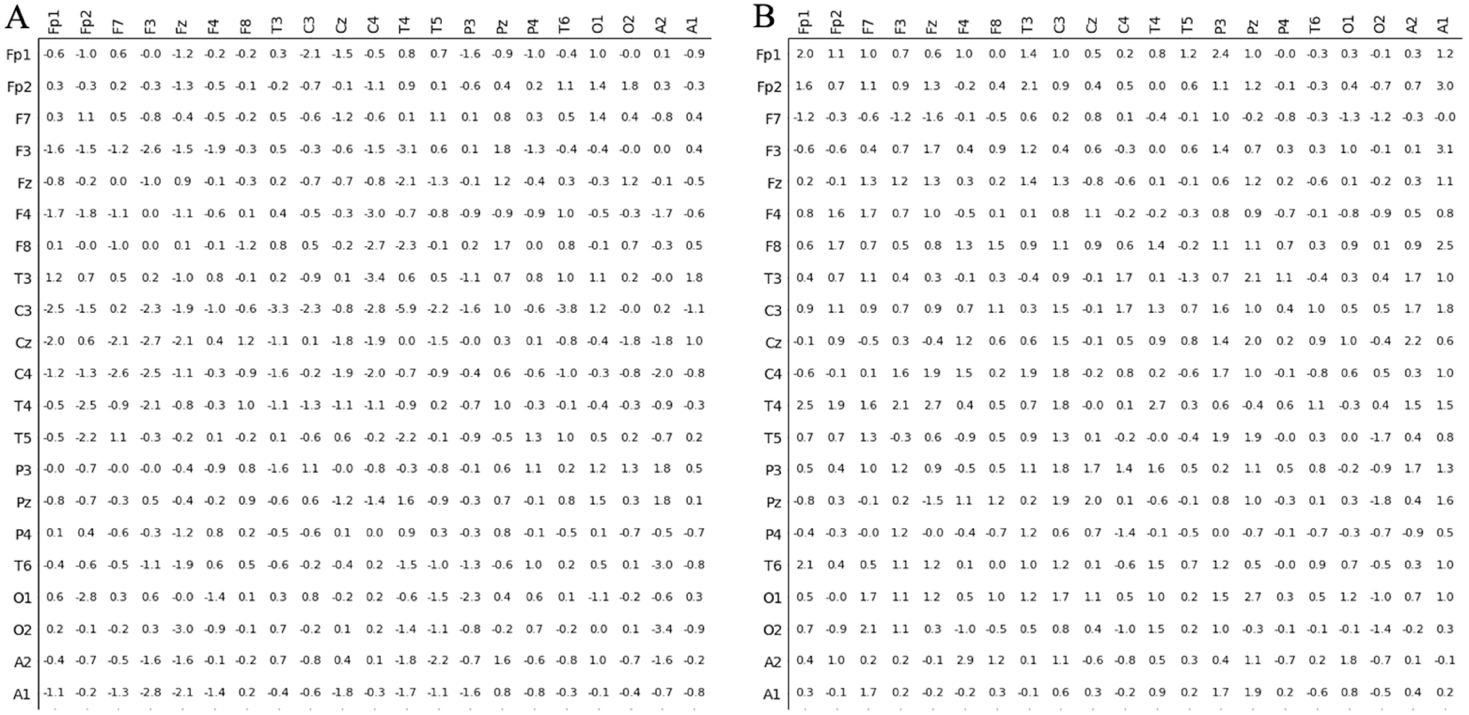


*Note*. The clusters found were not significant for either Theta band (all *p* > 0.15; all *p*_FDR_ > 0.30) or Alpha band (all *p* > 0.27; all *p*_FDR_ > 0.36).
